# Serum MicroRNA Expression Profile Distinguishes Enterovirus 71 and Coxsackievirus 16 Infections in Patients with Hand-Foot-and-Mouth Disease

**DOI:** 10.1371/journal.pone.0027071

**Published:** 2011-11-08

**Authors:** Lunbiao Cui, Yuhua Qi, Haijing Li, Yiyue Ge, Kangchen Zhao, Xian Qi, Xiling Guo, Zhiyang Shi, Minghao Zhou, Baoli Zhu, Yan Guo, Jun Li, Charles W. Stratton, Yi-Wei Tang, Hua Wang

**Affiliations:** 1 Institute of Pathogen Microbiology, Jiangsu Provincial Center for Disease Prevention and Control, Nanjing, China; 2 Department of Pathology, Vanderbilt University Medical Center, Nashville, Tennessee, United States of America; 3 Nanjing Children's Hospital, Nanjing, China; 4 Department of Medicine, Vanderbilt University Medical Center, Nashville, Tennessee, United States of America; University of Hong Kong, Hong Kong

## Abstract

Altered circulating microRNA (miRNA) profiles have been noted in patients with microbial infections. We compared host serum miRNA levels in patients with hand-foot-and-mouth disease (HFMD) caused by enterovirus 71 (EV71) and coxsackievirus 16 (CVA16) as well as in other microbial infections and in healthy individuals. Among 664 different miRNAs analyzed using a miRNA array, 102 were up-regulated and 26 were down-regulated in sera of patients with enteroviral infections. Expression levels of ten candidate miRNAs were further evaluated by quantitative real-time PCR assays. A receiver operating characteristic (ROC) curve analysis revealed that six miRNAs (miR-148a, miR-143, miR-324-3p, miR-628-3p, miR-140-5p, and miR-362-3p) were able to discriminate patients with enterovirus infections from healthy controls with area under curve (AUC) values ranged from 0.828 to 0.934. The combined six miRNA using multiple logistic regression analysis provided not only a sensitivity of 97.1% and a specificity of 92.7% but also a unique profile that differentiated enterovirial infections from other microbial infections. Expression levels of five miRNAs (miR-148a, miR-143, miR-324-3p, miR-545, and miR-140-5p) were significantly increased in patients with CVA16 versus those with EV71 (*p*<0.05). Combination of miR-545, miR-324-3p, and miR-143 possessed a moderate ability to discrimination between CVA16 and EV71 with an AUC value of 0.761. These data indicate that sera from patients with different subtypes of enteroviral infection express unique miRNA profiles. Serum miRNA expression profiles may provide supplemental biomarkers for diagnosing and subtyping enteroviral HFMD infections.

## Introduction

More than 500,000 hand-foot-and-mouth disease (HFMD) cases including 176 fatal cases have been reported occurred in China since March 2008 [1]. Human enterovirus 71 (EV71) and coxsackievirus A16 (CVA16) were the two major causative agents of HFMD accounting for more than 70% of cases in recent outbreaks [2]. Understanding the host responses following enteroviral infection should facilitate our understanding of enteroviral pathogenesis and may allow the development of diagnostic and treatment strategies. Host cellular response to enteroviral infections is an intricate process involving a number of molecular pathways. Genes involved in apoptosis, cellular development and differentiation, oncogenesis, and host transcription and translation are modified during enteroviral infections [3]. Several transcription factors such as E2F transcription factor 1, EIF3S9, and EIF2B5, are down-regulated early in the infection and then up-regulated later in the infection [3]. Other genes encoding antigen presentation molecules, immune cell activation markers, chemokines, and interferon (IFN)-inducible gene products are known to be modulated in response to viral infections [3], [4].

Host microRNA (miRNA) regulates cell processes by inactivating specific mRNAs via annealing to their 3′ untranslated region. Recently, miRNA has been noted to play a crucial role in the host cellular response to viral infections [5], [6]. For example, Ho and colleagues found that induced miR-141 following enteroviral infection contributed to down-regulation of host translation by targeting the translation initiation factor EIF4E [7]. Our previous study results showed that miRNAs are involved in neurological process, immune response, and cell death pathways, playing an important role in the host response to enteroviral infections [8]. Circulating miRNAs in serum or plasma are resistant to RNase and have a consistent expression level among individuals infected with the same species [9]. These unique characteristics of circulating miRNAs may provide a useful biomarker for supplemental diagnosis and prognosis.

Aberrant circulating miRNA expression has been reported in various non-infectious conditions [10], [11] as well as in infectious diseases such as sepsis [10], [11], HBV [12], [13], [14], and HCV [15]. In the present study, we evaluated serum miRNA expression levels following enteroviral infections, and whether these changes could be used to assist in detection these infections as well as discrimination of specific enteroviral subtypes causing these infections.

## Materials and Methods

### Research Subjects and Clinical Samples

Serum specimens included in the study were collected from pediatric patients with HFMD and other microbial infections as well as healthy children from May 2008 to April 2009. Healthy subjects were the children who received regular health check and absence of infections. Serum specimens collected from patients with other microbial infections including *Mycobacterium tuberculosis*, *Bordetella pertussis*, varicella-zoster virus, mumps virus, and measles virus were also collected contemporarily. Serum specimens from HFMD patients were collected within 72 h of the onset of clinical symptoms. All serum samples prior to miRNA analysis were stored at −80°C within 4 h following collection. Etiologic diagnosis of enteroviral infections in patients was confirmed by detection of EV71 or CVA16 from throat, stool or vesicle swab specimens using a duplex real-time reverse-transcriptive PCR [16]. This project was approved by the Ethics Committee of Jiangsu Provincial Center for Diseases Prevention and Control and written informed consent was obtained from parents or legal guardians of all children.

### Host miRNA Profiling Screening Using TaqMan Low-Density Array

A panel of host miRNAs were assessed using TaqMan Array Human miRNA Panel A+B (Applied Biosystems, CA), which detects 377 functionally defined miRNAs as well as 287 miRNAs whose function has not yet been completely defined. Total RNA was extracted from 400 µl of 20 pooled specimens using a mirVana PARIS kit (Ambion, Austin, TX) following the manufacturer's protocol for liquid samples. For miRNA amplification and detection, 1.67 µl RNA was reverse transcribed by using a TaqMan MiRNA Reverse Transcription Kit (Applied Biosystems) and 2.5 µl RT products were preamplified using Megaplex PreAmp Primers and regents. Megaplex RT reactions were diluted 150-fold with water, and 450 µl of each diluted product was combined with 450 µl of TaqMan 2× Universal PCR Master Mix (Applied Biosystems). One-hundred microliters of the sample/master mix for each Megaplex pool were loaded into the array, and the qRT-PCR was carried out on an Applied BioSystems 7900HT thermocycler using the manufacturer's recommended cycling conditions. Data were analyzed with SDS Relative Quantification Software version 2.3 (Applied BioSystems) and the threshold cycle (CT) values equal to or greater than 40 were treated as 40. A miRNA was considered altered if CT<35 in the one of the two groups and the miRNA showed ≥2-fold differences in concentration between the patient and control groups.

### Candidate miRNA Confirmation and Quantification

Total RNA from 200 µl of individual serum was extracted according to a method described previously [17]. Synthetic *C. elegans* miRNA (cel-miR-238, Takara Biotechnology Co, Dalian, China) was spiked-in each extracted RNA at a final concentration of 25 fmol as an internal control. Expression levels of candidate miRNA from the array analysis and spiked-in *C. elegans* miRNA were further confirmed and quantified by using the TaqMan miRNA Reverse Transcription Kit (Applied BioSystems). cDNA was obtained by using miRNA-specific stem-loop primers in a scaled down (5 µl) RT reaction (1.387 µl of water, 0.5 µl of 10× Reverse-Transcription Buffer, 0.063 µl of RNase-Inhibitor (20 units/µl), 0.05 µl of 100 mM dNTPs, 0.33 µl of Multiscribe Reverse Transcriptase, 1 µl RT primers and 1.67 µl sample RNA. RT reactions were performed using following conditions: 16°C for 30 min, 42°C for 30 min, 85°C for 5 min, hold at 4°C. Real time quantitative PCR reaction was performed in a final volume of 10 µl containing 4.5 µl diluted cDNA (1∶15), 5 µl TaqMan Universal PCR Master Mix (No AmpErase) and 0.5 µl TaqMan miRNA Assay (Applied BioSystems). The thermal cycle was set as start with 10 min template denaturation at 95°C, 40 cycles of denaturation at 95°C for 15 s and combined primer annealing/elongation at 60°C for 1 min. Each serum sample for each miRNA was run in triplicate.

### Statistical Analysis

For real-time PCR assays, expression levels of miRNAs were calculated using the CT values. Relative quantitation value of each miRNA was calculated by using the equation 2^−ΔCT^, in which ΔCT = CT_target miRNA_-CT_cel-miR-238_. Then the relative quantitation value of each miRNA underwent log2-transformation and employed to show the relatively expression levels of each target miRNAs [18], [19]. Statistical analysis was performed with SPSS software version 16.0 (SPSS, Inc., Chicago, USA). A *P* value<0.05 was considered statistically significant. For each miRNA, a receiver operating characteristic (ROC) curve was generated. Area under curve (AUC) value and 95% confidence interval (CI) were calculated to determine the specificity and sensitivity of enteroviral infection and subtype prediction. To increase the diagnostic accuracy of combination of changes in plasma miRNA levels, multiple logistic regression analysis were performed according to methods previously described [20].

## Results

During the study period, a total of 211 serum samples were collected in which 70 were from HFMD patients, 100 from other microbial infections, and 41 from healthy children. Among the 70 HFMD samples, 46 and 24 were confirmed to have EV 71 and CVA16 infections, respectively. There were no significant differences in age and gender distributions among EV71- and CVA16-infected patients and healthy children (**Table S1**).

In order to identify potential candidate miRNAs whose levels are significantly altered in response to enteroviral infection, the TaqMan Human miRNA Low density arrays (TLDA) analysis was performed. We screened serum miRNAs from patients with enteroviral infections in comparison to healthy controls. Of the 664 miRNAs incorporated in the array, 126 and 151 miRNAs were detected in serum of healthy controls and patients, respectively. Among them, 102 were found significantly elevated and 26 reduced in the samples from patients with specific enteroviral infection in relation to the levels detected in healthy controls (**Table S2**, details in **Table S3**). Ten miRNAs (miR-148a, miR-628-3p, miR-143, miR-324-3p, miR-206, miR-545, miR-140-5p, miR-597 miR-455-5p and miR-362-3p) that were significantly up-regulated (≥5 CT differences between the patient and control groups) were chosen for further analysis.

Analysis ten miRNAs expression profiles in six microbial infections (*M. tuberculosis*, *B. pertussis*, varicella-zoster virus, mumps virus, measles virus and enteroviruses) and healthy controls revealed that most miRNAs profiles varied among different microbial infections. The only exception is miR-140-5p, which was altered in every microbial infection in comparison to the healthy control (**Table 1**). The ten miRNAs expression profiles were unique for enteroviruses in comparison to other microbial infections (**Table 1**).

**Table 1:** CT values of ten miRNA candidates in serum pool prepared from 20 healthy controls and 20 patients with various microbial infections assessed by TaqMan Low-Density array.

miRNA	HC	EV	VZV	MEV	MUV	BP	MT
miR-148a	40	30.51	29.02	31.09	30.13	28.72	40
miR-143	40	30.99	40	32.76	31.46	40	40
miR-324-3p	38.23	32.66	32.99	34.63	29.85	40	34.87
miR-628-3p	33.43	28.27	34.74	31.86	35.66	30.35	30.53
miR-140-5p	40	29.24	27.86	28.51	29.81	26.69	30.13
miR-362-3p	40	32.38	40	40	33.06	40	40
miR-206	39.95	32.11	40	40	40	32.16	34.20
miR-455-5p	40	32.20	40	33.07	40	40	40
miR-597	40	32.63	33.96	40	32.36	40	40
MiR-545	40	32.32	40	40	40	40	40

HC:Healthy control; EV:enterovirus; VZV: varicella-zoster virus; MEV: measles virus; MUV: mumps virus; BP: *Bordetella pertussis*; MT: *Mycobacterium tuberculosis*.

**Figure 1:**
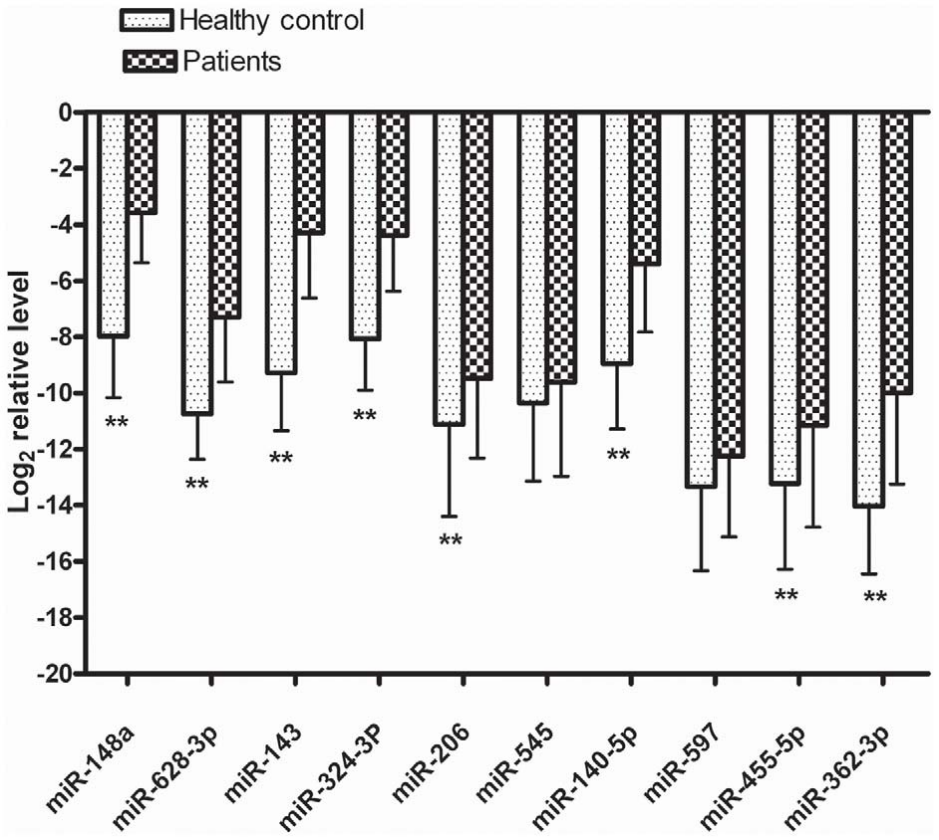
Differential serum expression levels of ten miRNAs in healthy control and patients with enterovirus infection. Serum levels of miR-140-5p, miR-143, miR-148a, miR-362-3p, miR-455-5p, miR-324-3p, miR-206, and miR-628-3p except for miR-545 and miR-597 were significantly higher in patients with enterovirus infection than those in the control group. **, *p*<0.01.

**Figure 2:**
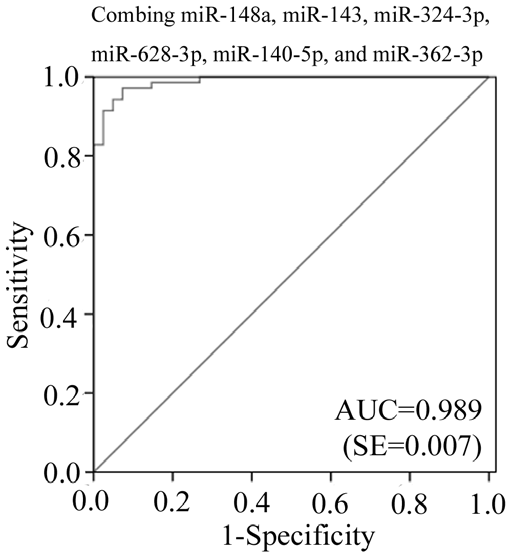
Receiver operating characteristic (ROC) curve for expression changed miRNAs to distinguish enterovirus infection from healthy control. Combination of miR-148a, miR-143, miR-324-3p, miR-628-3p, miR-130-5p, and miR-362-3p showed stronger distinguish efficiency. The AUC of 0.989 was obtained by combining above six miRNAs.

The relative levels of the ten randomly selected candidate mRNAs in individual samples were confirmed and determined using qRT-PCR. Serum levels of miR-148a, miR-628-3p, miR-143, miR-324-3p, miR-206, miR-140-5p, miR-455-5p and miR-362-3p were significantly higher in the sera of patients with enteroviral infections than in those from healthy controls. Expression differences of miR-545 and miR-597 between patients and healthy controls were not statistically significant (*p*>0.05) (**Figure 1**). ROC curves of miR-148a, miR-143, and miR-324-3p reflected strong separation between the enteroviral infection group and the healthy control group, with an AUC of 0.934, 0.931, and 0.901, respectively. The ROC curve of miR-628-3p, miR-140-5p, and miR-362-3p showed moderate ability to distinguish between the enteroviral infection group and the healthy control group, with an AUC of 0.877, 0.847, and 0.828, respectively (**Table 2**). Finally, the ROC curve of miR-206 and miR455-5p showed poor ability to distinguish between the enteroviral infection group and the healthy control group, with an AUC of less than 0.7 (Data not shown). At a cut-off point set at −4.94, miR-148a yielded a sensitivity of 81.2% and a specificity of 92.9%. At a cut-off point set at −6.35, miR-143 yielded a sensitivity of 87% and a specificity of 95.2%. At a cut-off point set at −6.30, miR-324-3p yielded a sensitivity of 85.5% and a specificity of 88.1% (**Table 2**). When expression levels of miR-148a, miR-143, miR-324-3p, miR-628-3p, miR-140-5p, and miR-362-3p were subjected to multiple logistic regression analysis, the resulting ROC curve had an AUC of 0.989 (95% CI 0.97–1.00) for correctly distinguishing enteroviral patients from healthy controls (**Figure 2**). At a cut-off point set at −16.15, this combination yielded a sensitivity of 97.1% and a specificity of 92.7%. The expression profile in combination of six miRNAs represented a unique fingerprint that differentiated enterovirial infections from other microbial infections.

**Table 2:** Areas under the receiver operating characteristic curve (AUC) and predictive value of six candidate miRNAs.

miRNA	AUC	95% CI	cut-off point	Sensitivity (%)	Specificity (%)
miR-148a	0.934	0.886–0.982	−4.94	81.2	92.9
miR-143	0.931	0.882–0.979	−6.35	87.0	95.2
miR-324-3p	0.901	0.838–0.964	−6.30	85.5	88.1
miR-628-3p	0.877	0.813–0.941	−8.66	73.9	92.9
miR-140-5p	0.847	0.773–0.920	−6.06	72.5	88.1
miR-362-3p	0.828	0.751–0.905	−12.07	81.2	81.0

**Figure 3:**
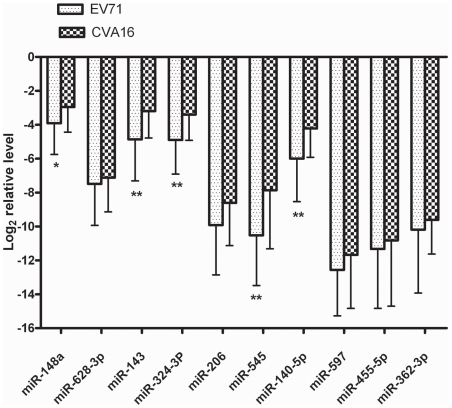
Differential serum expression levels of ten miRNAs in EV71 group and CVA16 group. Serum levels of miR-148a, miR-143, miR-324-3p, miR-545, miR-140-5p, were significantly higher in CVA16 group than those in the EV71 group. *, *p*<0.05. **, *p*<0.01.

**Figure 4:**
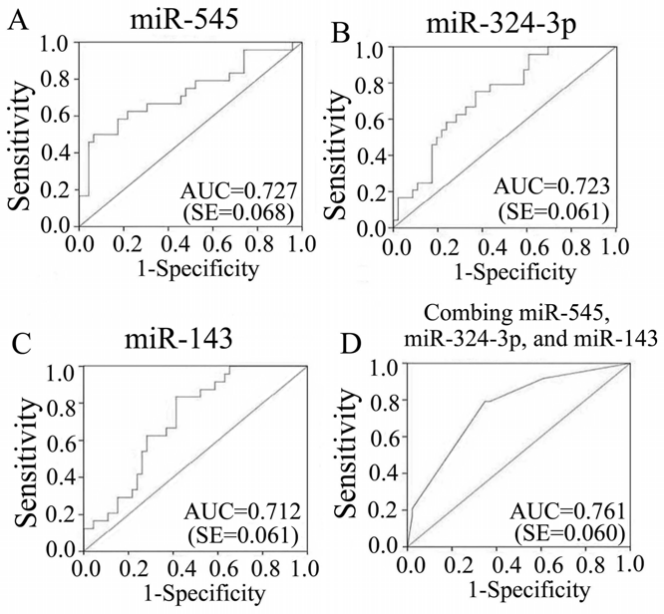
Receiver operating characteristic (ROC) curve for expression changed miRNAs to distinguish CVA16 infection from EV71 infection. ROC curve of miR-545 (A), miR-324-3p (B), and miR-143 (C) showed moderate distinguish efficiency. The AUC was mild increased to 0.761 through combining above three miRNAs (D).

To explore whether serum miRNA expression levels can differentiate enteroviral subtypes, the expression levels of above 10 miRNAs were further contrasted in patients infected with EV71 and CVA16. Five miRNAs including miR-148a, miR-143, miR-324-3p, miR-545, and miR-140-5p showed significantly higher expression in the CVA16 group when compared with the EV71 group (**Figure 3**). ROC curve analyses revealed that the serum levels of miR-545, miR-324-3p, and miR-143 showed moderate ability to differentiate patients with CVA16 infection from those with EV71 infection, with an AUC of 0.727 (95% CI = 0.594–0.860), 0.723 (95% CI = 0.603–0.843), and 0.712 (95% CI = 0.592–0.832). The ROC curve of miR-148a and miR-140-5p showed poor ability to distinguish between infection with CVA16 and infection with EV71, with an AUC of less than 0.7 (Data not shown). The AUC was slightly increased to 0.761 (95% CI = 0.644–0.879) by combining the above 3 miRNAs as described above (**Figure 4**). At the optimal cutoff value of 0.296, the sensitivity and specificity was 0.792 and 0.652, respectively.

## Discussion

Various laboratory methods have been developed to diagnose enteroviral infections. Isolation of virus followed by immunostaining with specific monoclonal antibodies is time consuming and labor intensive. Serological diagnosis, which is based on a fourfold rise of antibody titer, generally takes 10–14 days after the onset of illness. Reverse-transcriptive PCR techniques, which target the enterovirus 5′ conserved un-transcribed region, have become widely used in the laboratory diagnosis of enteroviral infections [16]. However, sub-optimally designed primer sets and inappropriate PCR procedures may result in false-negative results by missing certain enteroviral subtypes and mutants as well as false-positive results due to cross reactions with rhinovirueses.

The most commonly used molecular diagnostic methods for viral infections, such as RT-PCR, dependent on the genetic sequence of specific pathogens. The mutation of the virus gene may result in false negative results. Recently host responsive circulating miRNAs have been intensively studied as a new noninvasive marker for both diagnosis and prognosis. Most published studies have been focused on different types of cancer [10], [11]. Other studies have demonstrated dysregulated serum/plasma miRNA expression in non-cancerous processes such as oxidative liver injury, pregnancy, heart disease, diabetes, and tissue injuries [19], [21], [22], [23], [24], [25], [26], [27], [28], [29]. More recently, the role of miRNA in pathogen-host infectious interactions has been highlighted. Human miRNAs have important functions in viral replication and may be used by host cells to control viral infection. Virus encoded miRNAs appear to function in controlling viral replication, limiting antiviral responses, inhibiting apoptosis, and stimulating cellular growth [30]. Therefore, it is not surprising to find that host cell miRNA expression are altered after various pathogen infections [8], [31], [32], [33], [34], [35]. Consistent with those observations, aberrant serum/plasma miRNA expression also have been found in patients with various microbial infections [10], [11], [12], [13], [14], [15]. These results supported the concept that expression alteration of circulating miRNAs can be associated with a specific patho(physiological) state [24], [36].

In this study, we investigated expression levels of a panel of host miRNA in patients with enteroviral infections and in healthy controls. By the two-step screening and confirmation approach, we identified eight miRNA which were significantly up-regulated (CT value difference ≥5) in patients in comparison to controls. To evaluate the efficiency of these dysregulated miRNAs for diagnosing and subtyping enteroviral infections, ROC curves were constructed for each miRNA. Expression levels of six miRNAs (miR-148a, miR-143, miR-324-3p, miR-628-3p, miR-140-5p, and miR-362-3p) showed good ability to efficiently distinguish enteroviral infections from other microbial infections, with AUCs that ranged from 0.828 to 0.934. However, there was no single candidate miRNAs to distinguish enterovirial infections from other microbial infection and healthy controls. For example, expression of miR-140-5p was up-regulated in all detected microbial infections. A profile consisted of multiple host miRNA profiles is needed to well represent unique characteristics of each microbial infection. In our study, combination of six selected host miRNAs not only created a unique profile for enterovirial infections but also significantly increased the diagnosis efficiency with AUC of 0.989.

Early clinical manifestations of HFMD caused by EV71 and CAV16 are indistinguishable. However, it has been reported that EV71 is more likely than CAV16 to cause severe/fatal neurological diseases such as aseptic meningitis or encephalitis [37]. Early and rapid differentiation of enterovirus subtypes may benefit disease management and allow improved prognosis prediction. We found five miRNAs (miR-148a, miR-143, miR-324-3p, miR-545, and miR-140-5p) showed significantly higher expression in patients with CVA16 infection compared with those with EV71 infection. One noticeable finding of the present study was that combination of expression levels of three miRNAs (miR-545, miR-324-3p, and miR-143) provided moderate ability to differentiate between EV71 and CVA16 infections. Li and colleagues reported that a group of serum miRNAs accurately discriminated HBV cases from HCV cases despite identical clinical manifestations of hepatitis for both HBV and HCV infections [13]. While definitive diagnosis depends on organism-specific detection methods, our data suggested that host miRNA prolifes provide a supplemental biomarker for enteroviral differential diagnosis and genotyping at an early stage of infection. An extended study is being designed to enroll additional HFMD patient having a variety of enteroviral subtype infections; additional relevant host miRNA molecules will be investigated.

## Supporting Information

Table S1 **Demographic data of HFMD patients and healthy controls.**
(DOC)

Table S2 Altered miRNAs patterns in sera of patients with enterovirus infections determined in pooled serum specimens using TaqMan Low Density array *.
(DOC)

Table S3 **Results of TaqMan Low-Density array profiling of serum from patients with enteroviral infection and healthy controls.**
(XLS)
