# Supplementary material for: Serum MicroRNA Expression Profile Distinguishes Enterovirus 71 and Coxsackievirus 16 Infections in Patients with Hand-Foot-and-Mouth Disease
Source: PLoS One. 2011 Nov 8;6(11):e27071. doi: 10.1371/journal.pone.0027071 (PMC3210764; doi:10.1371/journal.pone.0027071)
Supplement: Table S1 — Demographic data of HFMD patients and healthy controls. (DOC) [file pone.0027071.s001.doc]

**Table S2.** Demographic data of HFMD patients and healthy controls

|  | Normal (N=41) | EV71 (N=46) | CVA16 (N=24) | p Value |
| --- | --- | --- | --- | --- |
| Gender, male (%) | 25 (61.0) | 26 (56.5) | 15 (62.5) | >0.05 |
| Age, years (mean±SD) | 2.66±2.70 | 3.56±2.00 | 2.57±1.60 | >0.05 |
