# Supplementary material for: Serum MicroRNA Expression Profile Distinguishes Enterovirus 71 and Coxsackievirus 16 Infections in Patients with Hand-Foot-and-Mouth Disease
Source: PLoS One. 2011 Nov 8;6(11):e27071. doi: 10.1371/journal.pone.0027071 (PMC3210764; doi:10.1371/journal.pone.0027071)
Supplement: Table S2 — Altered miRNAs patterns in sera of patients with enterovirus infections determined in pooled serum specimens using TaqMan Low Density array *. (DOC) [file pone.0027071.s002.doc]

**Table S2.** Altered miRNAs patterns in sera of patients with enterovirus infections determined in pooled serum specimens using TaqMan Low Density array *.

| ΔCT | Up-regulated (N=106) ** | Down-regulated (N=30) ** |
| --- | --- | --- |
| ≥5.0 | 37 | 7 |
| 2- | 41 | 14 |
| 1- | 28 | 9 |

* Expression of 536 miRNAs was unchanged or undetectable. See texts for definitions

** The 106 up-regulated and 30 down-regulated miRNAs included 4 duplicates, respectively. After removing the duplicates, there were total 102 up-regulated and 26 down-regulated miRNAs.
